# Effects of SDS on the activity and conformation of protein tyrosine phosphatase from *thermus thermophilus* HB27

**DOI:** 10.1038/s41598-020-60263-4

**Published:** 2020-02-21

**Authors:** Hai Hou, Huawei He, Yejing Wang

**Affiliations:** 1 State Key Laboratory of Silkworm Genome Biology, College of Biotechnology, Southwest University, Beibei, Chongqing 400715 China; 2 Institute of Medical Research, Northwestern Polytechnical University, Xi’an, Shaan Xi 710072 China; 3 Biological Science Research Center, Southwest University, Beibei, Chongqing 400715 China; 4 Chongqing Key Laboratory of Sericultural Science, Chongqing Engineering and Technology Research Center for Novel Silk Materials, Southwest University, Beibei, Chongqing 400715 China

**Keywords:** Transferases, Phosphorylases

## Abstract

Deciphering the activity-conformation relationship of PTPase is of great interest to understand how PTPase activity is determined by its conformation. Here we studied the activity and conformational transitions of PTPase from *thermus thermophilus* HB27 in the presence of sodium dodecyl sulfate (SDS). Activity assays showed the inactivation of PTPase induced by SDS was in a concentration-dependent manner. Fluorescence and circular dichroism spectra suggested SDS induced significant conformational transitions of PTPase, which resulted in the inactivation of PTPase, and the changes of α-helical structure and tertiary structure of PTPase. Structural analysis revealed a number of hydrophobic and charged residues around the active sites of PTPase may be involved in the hydrophobic and ionic bonds interactions of PTPase and SDS, which are suggested to be the major driving force to result in PTPase inactivation and conformational transitions induced by SDS. Our results suggested the hydrophobic and charged residues around the active sites were essential for the activity and conformation of PTPase. Our study promotes a better understanding of the activity and conformation of PTPase.

## Introduction

Protein reversible phosphorylation is one of the most important mechanisms in eukaryotes such as cell cycle, cell growth, proliferation, differentiation and migration^1,2^. Tyrosine phosphorylation and de-phosphorylation at cellular level are mainly regulated by protein tyrosine phosphatase (PTPase) and protein tyrosine kinase (PTKase) *in vivo*^3–5^. PTKase (EC 2.7.10.2) catalyze the phosphorylation of tyrosine residue, whereas PTPase (EC 3.1.3.48) catalyze the hydrolysis of a phosphorylated tyrosine residue to remove phosphate. PTPase is a superfamily of structurally diverse signal transduction enzyme^6,7^, which is classified into four sub-families: (1) pTyr- specific phosphatase; (2) dual specificity phosphatase; (3) cdc25 phosphatase; (4) low molecular weight (LMW) phosphatase^6^. All PTPases share a highly conserved motif or P-loop C(X)_5_R(S/T), of which Cys and Arg residues are essential for PTPase activity^8,9^. Various domains and subunits determine a variety of PTPase functions. PTPases are increasingly identified as potential targets for the related diseases^6,7,10^.

*Thermus thermophilus* is an extremely thermophilic bacterium, and has attracted great attentions for its scientific and economic value since its genomic sequence was resolved in 2004^11^. The crystal structure of Tt1001 (PDB: 2CWD), a LMW PTPase from *thermus thermophilus* HB8, has been resolved at 1.9 Å resolution in 2005. In 2009, we cloned PTPase gene from *thermus thermophilus* HB27, overexpressed and characterized the recombinant PTPase^12^. It is well-known the activity of an enzyme is closely dependent on its conformation. Although our knowledge on the structure and cellular function of PTPase is still increasing, little is known how the activity of PTPase from *thermus thermophiles* HB27 is determined by its conformation. Denaturant is usually used to characterize the activity and conformation of an enzyme^13–18^. Urea is known to break non-covalent interactions. It is more efficient at breaking hydrogen bonds than hydrophobic interactions^19,20^. Guanidinium chloride (GdnHCl) is a strong denaturant used in physiochemical study of protein folding. GdnHCl reduces the order degree of protein structure formed by water molecules, both in the bulk and the hydration shells surrounding the hydrophobic amino acids, thus resulting in protein denaturation. Sodium dodecyl sulfate (SDS) is an anionic surfactant with a long hydrophobic tail and a hydrophilic head^21,22^. In the presence of SDS, proteins interact with SDS to form negatively charged SDS-protein complex^23^. SDS could break the hydrophobic interactions, ionic bonds interactions and hydrogen bonds, while the disulfide bridges are not affected by SDS^24^.

We have investigated the unfolding of PTPase induced by urea and GdnHCl, and revealed two different unfolding pathways of PTPase in the presence of urea and GdnHCl in 2014^25^. Although SDS is neither implicated in the normal biological function of PTPase, nor is a ligand for PTPase, SDS is an effective denaturant to explore the conformation and activity of PTPase. Here, we used SDS to explore the activity and conformational transition of PTPase. Our result suggested the hydrophobic and electrostatic interactions between the residues around the active sites of PTPase and SDS were the major driving force to cause the inactivation and conformational transitions of PTPase. Therefore, the hydrophobic and charged residues around the active sites of PTPase are essential for the activity and conformation of PTPase. Our study facilitates a better understanding of PTPase conformation and activity.

## Results

### Effects of SDS on the activity of PTPase

**Figure 1:**
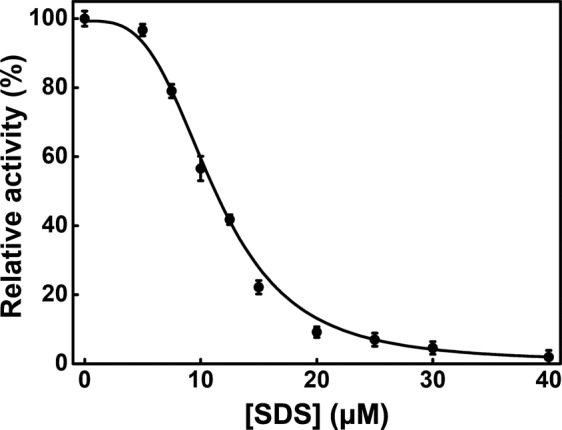
The relation of the relative residue activity of PTPase and SDS concentration.

To study the effects of SDS on the activity of PTPase, the residual activity of PTPase in the presence of 0–40 μM SDS was measured. The relationship of PTPase relative activity and SDS concentrations was determined, as shown in Fig. 1. The results showed the relative activity of PTPase declined gradually with the increase of SDS concentration. The relative activity of PTPase was 95% in the presence of 5 μM SDS. While increasing SDS concentration to 20 μM, the relative activity of PTPase decreased quickly from about 95% to 10%, indicating the conformation around the active sites of PTPase has been altered significantly by SDS. *IC*_50_ was estimated to be 11.27 μM by the extrapolation of the inactivation curve to x-axis. While further increasing SDS concentration more than 40 μM, PTPase activity continued to decrease slowly until it was abolished completely. The result suggested that SDS induced the conformational change of PTPase active sites in a concentration- dependent manner, thus resulting in the inactivation of PTPase.

### Intrinsic fluorescence spectra of PTPase in the presence of SDS

**Figure 2:**
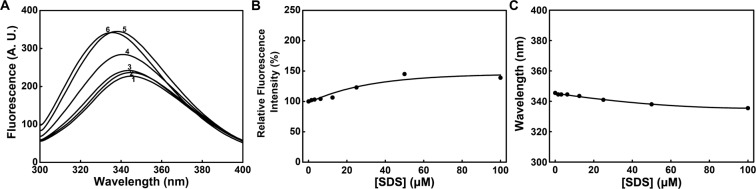
(**A**) Intrinsic fluorescence spectra of PTPase in the presence of 0–100 μM SDS. SDS concentrations for curves 1–6 were 0, 6.25, 12.5, 25, 50 and 100 μM, respectively. Relative changes of Imax (**B**) and λmax (**C**) *vs* SDS concentration.

To determine the effect of SDS on the tertiary structure of PTPase, the intrinsic fluorescence spectra of PTPase in the presence of 0–100 μM SDS were collected. The result showed not only the maximum fluorescence emission intensity (Imax), but also the maximum emission wavelength (λmax) of PTPase had been changed by SDS (Fig. 2A). In the presence of 0–12.5 μM SDS, the fluorescence spectra of PTPase (curves 1–3 in Fig. 2A) were slightly changed. While increasing SDS concentration from 12.5 to 100 μM, the fluorescence spectra of PTPase (curves 3–6 in Fig. 2A) were changed significantly while compared to that of native PTPase.

The relative changes of Imax and λmax of PTPase in the presence of SDS were plotted in Fig. 2B,C, respectively. The result showed Imax increased with the increase of SDS concentration from 0 to 100 μM, while compared to that of native PTPase. Imax increased not more than 10% in the presence of 0–12.5 μM SDS. While increasing SDS concentration to 50 μM, Imax increased about 40%. As shown in Fig. 2C, SDS induced a progressive blue-shift of λmax from 345 to 338 nm while gradually increasing SDS concentration from 0 to 50 μM. With further increasing SDS concentration to 100 μM, λmax continued to blue-shift from 338 to 335.5 nm.

### ANS fluorescence spectra of PTPase in the presence of SDS

**Figure 3:**
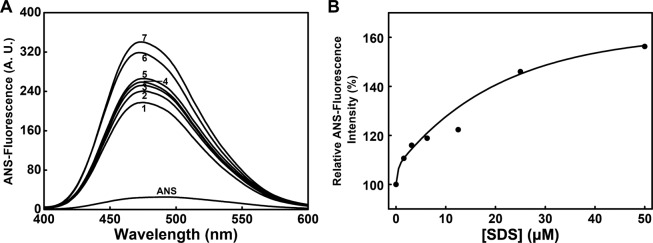
(**A**) ANS fluorescence spectra of PTPase in the presence of 0–50 μM SDS. SDS concentrations for curves 1–7 were 0, 1.56, 3.125, 6.25, 12.5, 25 and 50 μM, respectively. (**B**) Relative changes of Imax *vs* SDS concentration.

To study the effects of SDS on the hydrophobic patches of PTPase, ANS fluorescence spectra of PTPase in the presence of 0–50 μM SDS were collected. The result showed ANS itself had a very low level of fluorescence with λmax at 492 nm. Once binding with the hydrophobic patches of PTPase, λmax immediately blue-shifted to 476 nm. Also, Imax increased about 10-fold compared to that of free ANS (Fig. 3A). While increasing SDS concentrations to 50 μM, λmax slightly blue-shifted to 472.5 nm. In addition, Imax increased with the increase of SDS concentration (Fig. 3A). The relation of ANS fluorescence intensity and SDS concentrations was shown in Fig. 3B. Compared to that of native PTPase, Imax increased about 20% in the presence of 12.5 μM SDS. While increasing SDS concentration to 25 μM, Imax increased about 45%. In the presence of 50 μM SDS, Imax increased about 55%. These results suggested that SDS induced significant conformational transitions of PTPase, thus resulted in the exposure of the buried hydrophobic residues of PTPase.

### Far-UV circular dichroism spectra of PTPase in the presence of SDS

**Figure 4:**
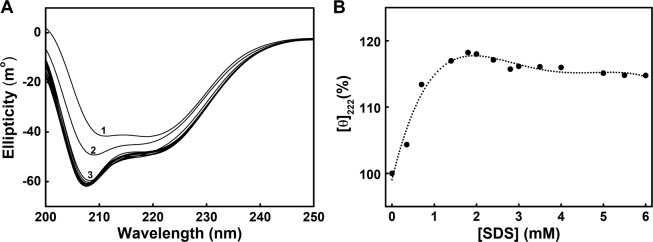
(**A**) Far-UV CD spectra of PTPase in the presence of 0–6 mM SDS. SDS concentrations for curves from top to bottom were 0, 0.35, 0.70, 1.40, 1.80, 2.00, 2.40, 2.80, 3.00, 3.50, 4.00, 5.00, 5.50 and 6.00 mM, respectively. (**B**) Relative changes of the elipticity at 222 nm *vs* SDS concentration.

To reveal the effects of SDS on the secondary structures of PTPase, far-UV circular dichroism (CD) spectra of PTPase in the presence of 0–6 mM SDS were collected. The result showed the ellipticity of 222 nm (θ_222_) increased with increasing SDS concentration from 0 to 0.7 mM (Fig. 4A), indicating that SDS induced the formation of PTPase α-helical structure. θ_222_ increased about 18% in the presence of 1.8 mM SDS (Fig. 4B), indicating that SDS induced a significant conformational transition of PTPase, thus resulted in the increase of PTPase α-helical contents. While further increasing SDS concentration to 6 mM, θ_222_ decreased slightly about 3.5%, indicating a small portion of α-helixes of PTPase were likely induced by SDS to transform into other secondary structures.

### Structural analysis

**Figure 5:**
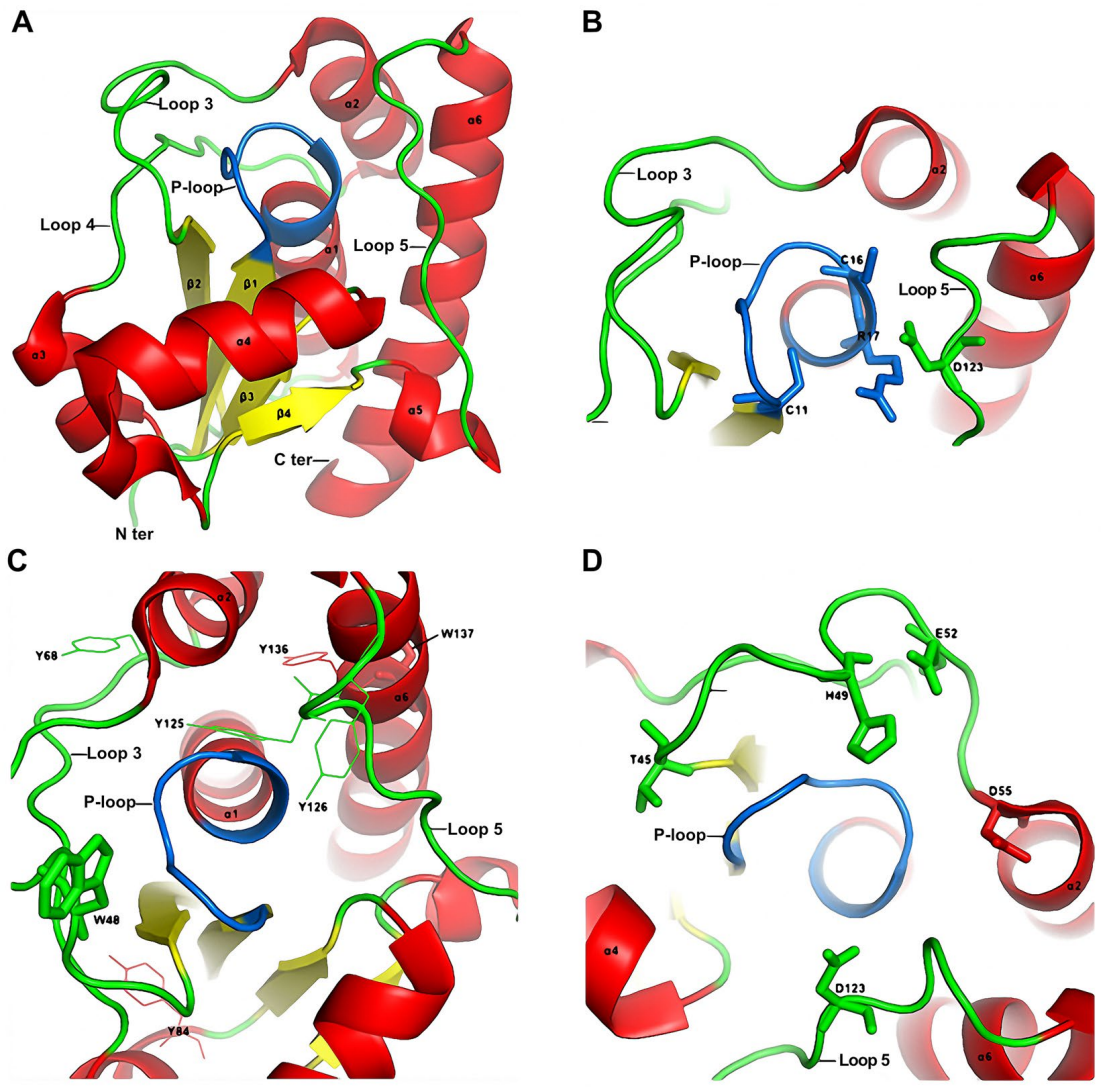
(**A**) Cartoon structure of Tt1001 protein. α-helix, β-sheet and loops were colored in red, yellow and green, respectively. P-loop was highlighted in marine. (**B**) Essential residues Cys11, Cys16, Arg17 and Asp123 were showed as sticks. (**C**) Tryptophan and tyrosine residues were showed as sticks and lines, respectively. (**D**) Polar and charged residues located on loop 3 and loop 5 are showed as sticks. All the structures are prepared by PyMOL (DeLano, Warren L., The PyMOL Molecular Graphics System (2008). DeLano Scientific, California, USA).

Tt1001 from *thermus thermophilus* HB8 is same as PTPase of *thermus thermophilus* HB27, as they have 100% sequence identity^25^. To better understand the effects of SDS on PTPase activity and conformation, here we analyzed the structure of Tt1001. Tt1001 contains the signature motif P-loop consisted of the active-site sequence **CLGNICRS** (Fig. 5A). All the amide protons between Cys11 and Ser18 face the center of P-loop, and they are well positioned for the binding of PTPase with phosphate (Fig. 5A,B). Among of these residues, Cys11 and Cys16 are essential for the activity of PTPase. Arg17 also plays an important role for the substrate binding with PTPase (Fig. 5B)^26–28^. It is notable there is a crevice leading from one side into the active sites of Tt1001. The variable loop 3 links β2 and α2, which is adjacent to P-loop and the active sites. The variable loop 3 partially interacts with P-loop, thus stabilizing the conformation of PTPase active sites. The loop 5 connects α5 and α6, which also contains a critical residue Asp123 for PTPase activity (Fig. 5A,B).

As shown in Fig. 5C, Trp48, Trp137 and most of tyrosine residues are located on the surface of PTPase and exposed to solvents. Such exposure may lead to a lack of constraint of these residues in PTPase^29^. Among of the exposed aromatic residues, Trp48, Tyr125, and Tyr126 line around the crevice and near the active sites of PTPase. The side chains of these residues form a relatively hydrophobic microenvironment, which is favorable for the entrance and binding of phosphor-tyrosine with the active sites of PTPase (Fig. 5C)^26,28^. In addition, some polar and charged residues such as Thr45, His49, Glu52 are located on the loop 3 of PTPase and exposed to solvents. The side chains of these residues are positioned to face P-loop, which are important to maintain the conformation of PTPase active sites (Fig. 5D).

## Discussion

Urea, GdnHCl and SDS are common chemical denaturants used to study the conformation-activity relation of an enzyme, however, they act in different ways. As a polar non-electrolyte, urea could form hydrogen bonds with peptide backbone, charged groups and water molecules in solution. The hydrogen bonds formation ability of urea is twice of water^21,30^. Hence, it is more efficient at breaking hydrogen bonds than hydrophobic interactions^19,20^. GdnHCl is able to reduce enzyme activity and improve protein molecules solubility. As a strong chaotrope, GdnHCl destroys the hydrogen bonds network of water and protein molecules, thus affecting the stability and activity of proteins in solution. Different from urea and GdnHCl, SDS consists of a 12-carbon tail and an anionic sulfate group, which endow SDS amphiphilic properties^21,22^. SDS interacts with proteins to form a negatively charged SDS-protein complex, thus disrupting the non-covalent interactions of protein chains^23^. Hence, SDS is widely applied in protein structural study to break the hydrophobic interactions, ionic bonds and hydrogen bonds^22,24,31–33^.

Our previous research has identified two different unfolding mechanism of PTPase in the presence of urea and GdnHCl. Urea and GdnHCl induce the unfolding of PTPase to form a partially active intermediate and an inactive intermediate, respectively^25^. Structural analysis, intrinsic and ANS fluorescence reveal that SDS also induces a compact denatured state like a molten globule state that has no significant change in the native secondary structure. The difference reflects the different mechanism of denaturants, which has been discussed in detail before. Urea (≤2.0 M) or GdnHCl (≤0.5 M) induces significantly a conformational transformation of PTPase, thus resulting in the loss of PTPase activity, the increase of α-helix structure and the formation of molten globule-like intermediate. Similarly, SDS (≤2.0 mM) induced the conformational change of PTPase active sites, decreased PTPase activity and increased PTPase α-helix content. The minimum concentrations required for the complete inactivation of PTPase for urea, GdnHCl and SDS are 8 M, 0.8 mM and 0.04 mM, respectively. The results suggest SDS is more efficient to inactivate PTPase activity than urea and GdnHCl, as SDS not only disrupts the hydrophobic interactions, but also breaks the hydrogen bonds and ionic bonds of PTPase.

Tryptophan residue usually has a maximal fluorescence emission wavelength at 340–350 nm when exposed to solvents^34–36^. The intrinsic fluorescence λmax of native PTPase was 345.5 nm (Fig. 2A), suggesting the tryptophan residues of PTPase likely exposed to solvents, which is consistent with the position of Trp48 and Trp137 in Tt1001 (Fig. 5C). In the presence of tryptophan residue, tyrosine fluorescence is often negligible due to their significant fluorescence ability differences. However, we noticed some slight changes in the intrinsic fluorescence spectra between 300 and 310 nm (curves 3–6 in Fig. 2A), which may come from the fluorescence emission of the exposed tyrosine residues^37^. As shown in Fig. 2B,C, λmax blue-shifted about 10 nm along with the increase of Imax in the presence of 100 μM SDS, indicating these exposed tryptophan and tyrosine residues of PTPase probably were induced by SDS into a relatively hydrophobic microenvironment^38^. This transition may arise from the hydrophobic interactions between the tryptophan and tyrosine residues of PTPase and the aliphatic chain of SDS. Morin is known to be easily extracted into SDS micelles through hydrophobic interaction^39^. Imax decreased with the increase of SDS concentration, which may be due to the fluorescence quenching caused by high concentration SDS^37^.

The ANS fluorescence spectra of PTPase showed that λmax blue-shifted and Imax increased along with the increase of SDS concentration (Fig. 3A,B), indicating more and more ANS bound with the hydrophobic residues of PTPase^40^. The exposure of the hydrophobic residues probably arose from the conformational transition induced partially by the hydrophobic interactions of PTPase and SDS^41^. As shown in Fig. 5, the hydrophobic residues are located on the flexible loops, which are near the active sites of PTPase and may be involved in the interactions of PTPase with SDS.

The far-UV CD spectra of PTPase suggested SDS induced the formation of α-helical structure (Fig. 4B)^42,43^. In addition to the hydrophobic interactions, the ionic bonds interactions between the anionic “head” of SDS and the exposed charged residues of PTPase such as Thr45, His49, Glu52 and Asp55 may also be involved in the conformational transitions of PTPase induced by SDS (Fig. 5D). The hydrophobic and ionic bonds interactions between SDS and PTPase probably induced the conformational transitions of PTPase, thus resulted in the formation of PTPase α-helix structure. In our previous study, we have discussed the effect of the charged residues around the active sites on PTPase activity. The ionic bonds interactions between Gdn^+^ and the charged residues disrupt the conformation of PTPase active sites, resulting in the inactivation of PTPase^25^. Here, our result again suggested the hydrophobic and charged residues around the active sites are essential for the conformation and activity of PTPase.

Based on the structure of Tt1001 and our findings, we suggest the flexible loops of PTPase, especially loop 3 and loop 5, contain a number of essential residues for the conformation of the active sites and substrate binding of PTPase. The hydrophobic and charged residues in the loops may interact with SDS *via* the hydrophobic and electrostatic interactions to induce the conformational changes of the loops, thus resulting in a slight decrease of PTPase activity in the presence of 0–5 μM SDS (Fig. 1). With the increase of SDS concentration and the conformational changes of these loops, the conformation of P-loop was gradually induced to expose to solvents, which possibly led to a direct interaction of P-loop with SDS. The interactions of P-loop and SDS further altered the conformation of PTPase active sites, resulting in the rapid loss of PTPase activity in the presence of 5–20 μM SDS. While further increasing SDS concentration, PTPase activity continued to decrease until the conformation of PTPase active sites was changed completely (Fig. 1). Meanwhile, the variable loops or other structures of PTPase were possibly induced by SDS to transform into α-helical structure (Fig. 4). These conformational transitions caused the exposure of the hydrophobic residues such as Trp48, Tyr125 and Tyr126 buried in the interior of PTPase, thus determined the intrinsic and ANS fluorescence spectra changes (Figs. 2, 3).

Although we have discussed the conformational transitions and the inactivation of PTPase induced by SDS in detail, more experimental evidences such as the complex structure of PTPase-SDS and the dynamics of PTPase-SDS interactions are still required to clarify the details of molecular interaction. Our study is valuable toward the long-term goal to better understand the activity and conformation of PTPase.

## Materials and Methods

### Reagents and materials

All chemicals used in this research such as *para*-nitrophenyl phosphate (*p*NPP), Isopropyl-β-D-1-thiogalactopyranoside (IPTG), Dithiothreitol (DTT), SDS and 1-anilinonaphtalene-8-sulfonate (ANS) were of the highest purity commercially available. *PTPase* from *thermus thermophilus* HB27 was cloned into pET-28a (+) vector (Novagen, Germany) and overexpressed in *E. coli* BL21 (DE3). PTPase was purified as described^12^. The concentration of recombinant PTPase was determined by BCA protein assay kit (Pierce, USA).

### PTPase activity assay

PTPase activity was assayed as described previously^25,44^. Briefly, *p*NPP (10 mM) and PTPase (2.4 μM) were added into 200 µL, 50 mM acetic acid-sodium acetate buffer (pH 3.8) plus 5 mM DTT. After incubation at 30 °C for 10 min, NaOH (1 M, 1 mL) was added into the mixture to terminate the reaction. The absorption change at 405 nm was recorded on a Helios-γ UV-VIS spectrophotometer (Thermo Scientific, USA). The residual activities of PTPase were measured after incubation of PTPase with variable concentrations of SDS at 25 °C for 2 h.

### Intrinsic and ANS fluorescence spectra

The intrinsic fluorescence spectra of PTPase in the presence of SDS were recorded on a F-2500 fluorescence spectrophotometer (Hitachi, Japan) at 25 °C, which is equipped with a water-circulating bath with an accuracy ± 0.10 °C. The intrinsic fluorescence spectra were excited at 280 nm and recorded from 300 and 400 nm using a 1 cm path-length quartz cuvette. ANS was used as a fluorescent probe to study the conformational changes of PTPase in the presence of various concentrations SDS. After addition of 10-folds molar excess of ANS into PTPase in the presence of 0–50 μM SDS, the samples were incubated at 25 °C for 30 min in dark prior to the tests. The ANS fluorescence emission spectra were excited at 380 nm and collected from 400 to 600 nm at 25 °C. All fluorescence spectra of PTPase were calibrated by subtraction of the fluorescence of SDS under the same experimental condition. Final spectra were the average of three corrected spectra. PTPase concentration was 2.4 μM for all the intrinsic and ANS fluorescence experiments.

### Far-UV CD spectra

The far-UV CD spectra of PTPase in the presence of SDS were recorded on a J-715 spectrophotometer (Jasco, Japan) at 25 °C with a thermostatically controlled cell holder attached to a water bath with an accuracy ± 0.10 °C. The CD spectra were collected from 250 to 190 nm using a 1 cm path-length quartz cuvette. Five spectra were collected for each sample and averaged after subtraction the signal of SDS under the same condition. The final concentration of PTPase was 11 μM for all CD experiments.
